# Developing Mixed Matrix Membranes with Good CO_2_ Separation Performance Based on PEG-Modified UiO-66 MOF and 6FDA-Durene Polyimide

**DOI:** 10.3390/polym15224442

**Published:** 2023-11-17

**Authors:** Kavya Adot Veetil, Asmaul Husna, Md. Homayun Kabir, Insu Jeong, Ook Choi, Iqubal Hossain, Tae-Hyun Kim

**Affiliations:** 1 Organic Material Synthesis Laboratory, Department of Chemistry, Incheon National University, Incheon 22012, Republic of Korea; kavya14396@gmail.com (K.A.V.); shopnilchem@gmail.com (A.H.); kabirchem07@gmail.com (M.H.K.); jis_3088@naver.com (I.J.); choiook@inu.ac.kr (O.C.); iqubal.chem.ru.08@gmail.com (I.H.); 2 Research Institute of Basic Sciences, Core Research Institute, Incheon National University, 119 Academy-ro, Yeonsu-gu, Incheon 22012, Republic of Korea

**Keywords:** gas separation membrane, CO_2_ separation, metal–organic framework, mixed matrix membrane, PEG-grafted MOF

## Abstract

The use of mixed matrix membranes (MMMs) comprising metal–organic frameworks (MOFs) for the separation of CO_2_ from flue gas has gained recognition as an effective strategy for enhancing gas separation efficiency. When incorporating porous materials like MOFs into a polymeric matrix to create MMMs, the combined characteristics of each constituent typically manifest. Nevertheless, the inadequate dispersion of an inorganic MOF filler within an organic polymer matrix can compromise the compatibility between the filler and matrix. In this context, the aspiration is to develop an MMM that not only exhibits optimal interfacial compatibility between the polymer and filler but also delivers superior gas separation performance, specifically in the efficient extraction of CO_2_ from flue gas. In this study, we introduce a modification technique involving the grafting of poly(ethylene glycol) diglycidyl ether (PEGDE) onto a UiO-66-NH_2_ MOF filler (referred to as PEG-MOF), aimed at enhancing its compatibility with the 6FDA-durene matrix. Moreover, the inherent CO_2_-philic nature of PEGDE is anticipated to enhance the selectivity of CO_2_ over N_2_ and CH_4_. The resultant MMM, incorporating 10 wt% of PEG-MOF loading, exhibits a CO_2_ permeability of 1671.00 Barrer and a CO_2_/CH_4_ selectivity of 22.40. Notably, these values surpass the upper bound reported by Robeson in 2008.

## 1. Introduction

Greenhouse gas emissions, particularly CO_2_ emissions, have escalated unprecedentedly primarily due to the continuous utilization of fossil fuel-based energy sources. Consequently, these energy sources are frequently identified as the primary contributors to the elevation of global temperatures and the ensuing changes to the climate. In response to this challenge, substantial efforts have been directed globally toward decreasing the CO_2_ concentration of the atmosphere. Among these efforts is the development of carbon capture, utilization, and storage (CCUS) technology. This technology focuses on capturing the CO_2_ generated from fossil fuel use and converting it into valuable substances, such as alcohols and bioplastics, thereby transforming CO_2_ emissions into a resource rather than a pollutant [1,2]. 

Presently, CO_2_ separation is predominantly accomplished through various methods including chemical absorption, cryogenic distillation, adsorption, and chemical looping. Simultaneously, considerable attention has been directed toward gas separation utilizing polymer membranes. This approach is favored due to its straightforward procedural nature and relatively modest initial installation expense compared to alternative CO_2_ separation methodologies. Additionally, this technique stands out not only for its compatibility with broad-scale implementations but also for its notable energy efficiency [1,3,4].

Despite the creation of numerous commercial polymer membrane materials in recent years, enhancing the permeability and selectivity of such membranes has proven challenging. This predicament arises due to an inherent trade-off relationship between these two parameters. In particular, polymer membranes possessing elevated permeability often display diminished selectivity; the converse is also true. This technical constraint is often illustrated using the well-known “Robeson’s plot” [5,6]. In light of this situation, exhaustive research endeavors have been undertaken to engineer separation membrane materials that can transcend the limitations imposed by the Robeson upper bound. 

Polyimide (PI)-based rigid polymer membranes, in particular, are used widely in gas separation applications due to their elevated glass-transition temperature (*T*_g_), exceptional thermal stability, and robust mechanical attributes. However, these membranes, characterized by their rigid polymer backbone, have relatively modest permeability. Moreover, they are susceptible to the “aging effect”, during which their permeability deteriorates gradually due to a reduction in their fractional free volume with time [4,7]. 

Concurrently, there has been a surge in research focused on gas separation involving porous materials with varying pore sizes, including zeolites [8,9], metal–organic frameworks (MOFs) [10,11,12,13], covalent organic frameworks (COFs) [14,15,16], and porous organic polymers [17,18]. By incorporating these porous materials as additives into organic polymer membranes, a class of materials known as mixed matrix membranes (MMMs) can be fabricated. Renowned for their elevated permeability and selectivity, MMMs also exhibit excellent mechanical attributes and chemical stability [19,20].

Among these materials, MOFs stand out as three-dimensional porous structures constructed through coordinated covalent bonds between metal atoms and organic molecules. MOFs have exceptional thermal stability, substantial free volume, specific surface area, intrinsic molecular-level porosity, and straightforward surface modification or functionalization. MOF-based MMMs combine the benefits of MOFs with the attributes of a polymer matrix, including outstanding mechanical properties and cost-effective processing. Nevertheless, precise control over the pore size of MOFs remains challenging. Furthermore, surpassing a certain threshold of MOF content can lead to non-uniform distribution within the polymer matrix, resulting in aggregation. Aggregation-induced defects in gas separation membranes significantly compromise their overall separation efficiency, posing a critical obstacle to their practical use [10,21].

In response, a variety of MOF-functionalization methods have been proposed. These techniques enhance the compatibility between MOFs and organic polymer membranes while elevating their gas separation performance [11,22,23,24]. Among these approaches, techniques involving replacing the metallic ions in the MOF with alternate metallic metals or the modification of organic linkers have been explored to regulate pore size and volume [25,26,27]. These strategies aim to selectively heighten the permeability or selectivity of specific gases. In parallel, other investigations have focused on augmenting the gas separation efficiency of membranes by covering the surface of MOF particles with either polymeric substances, such as poly(vinyl alcohol) (PVA) [28], or surface-active agents [29,30,31]. These coatings should improve the interaction between the MOF particles and the polymer matrix, enhancing dispersibility and interfacial adhesion. 

Furthermore, previous research conducted by the authors of this study has involved coating MOF particle surfaces with diverse functional groups, including amino, carboxyl, and hydroxyl groups [11,22,23]. These coatings enhanced the interaction between the particles and the organic polymer membranes, improving gas separation performance. Additionally, organic oligomers were introduced into these functional groups to amplify not only gas permeability and selectivity but also their compatibility with polymer membranes. Thus, the introduction of organic oligomers was intended to counteract MOF particle aggregation. 

Polymers incorporating ethylene oxide (EO) units, such as polyethylene glycol (PEG), have found extensive applications in the production of gas separation membranes owing to their pronounced CO_2_ solubility. Nevertheless, when the EO unit concentration in polymers surpasses a specific threshold, the molecules tend to aggregate due to heightened crystallinity, causing a decline in permeability [32].

We have recently reported the synthesis of PIM-[durene(m)-co-PEG/PPG(n)]-polyimide membranes. These membranes contained a rigid porous polymer unit with intrinsic microporosity (PIM) and a soft PEG/PPG unit that were uniformly mixed due to a unique chain threading effect [33]. This uniform chain threading of PEG/PPG units onto the PIM structure resulted in well-mixed PEG/PPG and PIM. However, incorporating the PEG/PPG unit greatly reduced the permeability of CO_2_ compared to that of pristine PIM, as the unique chain threading effect disturbed the free volume of the highly porous PIM.

In this work, Zr-based UiO-66-NH_2_, notable for its permeability, was subjected to grafting with PEG, yielding a PEG-grafted MOF. As a filler, the PEG-modified UiO-66-NH_2_ (PEG-UIO-66-NH_2_) was then incorporated in a 4,4′-(Hexafluoroisopropylidene) diphthalic anhydride-durene polyimide (6FDA-durene PI), used as the matrix or support, to prepare a self-standing MMM for high-performance CO_2_ separation. The effect of the PEG-modified MOF filler loading levels relative to 6FDA-durene PI on the thermal behavior, morphology, and gas separation behavior was then extensively investigated.

Polyethylene glycol diglycidyl ether (PEGDE)-modified UiO-66-NH_2_ (PEG-UIO-66-NH_2_) has been previously crosslinked with polyvinyl amine (PVAm) and used to coat a polysulfone substrate and prepare a composite membrane [34]. The ether oxygen groups derived from PEG-UIO-66-NH_2_, in conjunction with the amino groups of PVAm, showed an enhanced affinity toward carbon dioxide (CO_2_). Although this work has demonstrated that PEG-modified MOF (PEG-MOF) can enhance CO_2_ separation performance, it differs from the current study because PEG-MOF was used as a coating material. The use of PEG-MOF as a filler in MMMs has not previously been reported.

## 2. Materials and Methods

### 2.1. Materials

4,4′-(Hexafluoroisopropylidene) diphthalic anhydride (6FDA), 2,3,5,6-tetramethylbenzene-1,4-diamine (durene), acetic acid (96%), and triethylamine were procured from Tokyo Chemical Industry (TCI) Co., Ltd. (Tokyo, Japan) and utilized as received. Acetic anhydride and poly(ethylene glycol) diglycidyl ether (PEGDE) were sourced from Sigma Aldrich (Yongin, Republic of Korea). Dimethyl acetamide (DMAc), methanol, and chloroform were obtained from Daejung Chemical and Metal Co. Ltd., Shiheung, Republic of Korea. Zirconium chloride (ZrCl_4_) and 2-amino terephthalic acid were acquired from Alfa Aesar (Seoul, Republic of Korea). Prior to polymerization, 6FDA was subjected to overnight heating at 150 °C, and durene was recrystallized in methanol and subsequently dried under vacuum at 40 °C for 24 h. Unless otherwise specified, all other chemicals were employed as received from commercial suppliers. 

### 2.2. Preparation of PEG-Functionalized UiO-66 MOF

The UiO-66-NH_2_ MOF was synthesized following the reported procedure [35]. In a 30 mL volume of dimethylformamide (DMF), a mixture containing ZrCl_4_ (0.50 g, 2.14 mmol), 2-amino terephthalic acid (0.39 g, 2.14 mmol), and 3.3 mL of acetic acid was dissolved. This solution was then placed in a Teflon-lined autoclave and heated at 120 °C in an oven. After heating, the resulting yellow suspension was centrifuged and subjected to multiple methanol washing steps. Further purification involved re-dispersing in methanol for a week followed by centrifugation. Subsequently, activation was carried out under vacuum at 120 °C for 24 h.

**Scheme 1:**
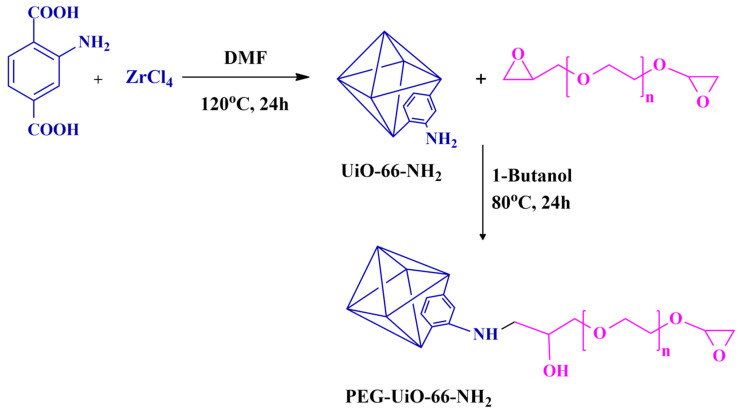
Synthesis of UiO-66-NH_2_ and PEG-MOF, a PEGDE-grafted MOF.

For the synthesis of PEG-UIO-66-NH_2_, a post-synthetic modification of UiO-66-NH_2_ was conducted as outlined in Scheme 1. Initially, UiO-66-NH_2_ nanoparticles (1.18 g) were dispersed in 1-butanol (74 mL) through sonication. Following dispersion, the solution was transferred to a two-neck round bottom flask, and an excess of PEGDE was dissolved in the suspension and stirred for 30 min. The modification was then carried out under reflux at 80 °C for 24 h under a N_2_ atmosphere. To eliminate excess unreacted PEGDE, the resultant PEG-UiO-66-NH_2_ underwent a series of steps, including centrifugation, dispersion, and ultrasonication, to exchange the solvent with fresh methanol. The final yellow powder product was dried at 50 °C under vacuum for 24 h, yielding the PEG-UIO-66-NH_2_ material. 

### 2.3. Synthesis of 6FDA-Durene Polyimide

The synthesis of 6FDA-durene polyimide was carried out using a general two-step procedure involving the initial formation of polyamic acid followed by imidization, as outlined below: 6FDA (2.49 g, 5.63 mmol), durene (0.93 g, 5.63 mmol), and DMAc (50 mL) were combined in a two-neck round bottom flask equipped with a nitrogen inlet and a condenser. The reaction mixture was cooled in an ice bath for 3 h, followed by raising the temperature to room temperature and stirring for 12 h to form poly(amic acid). To induce imidization, triethylamine (1.65 mL) and acetic anhydride (0.74 mL) were introduced into the reaction mixture. After the addition, the temperature was elevated to 110 °C, and the reaction mixture was vigorously stirred for 3 h. The resulting viscous polymeric solution was cooled to room temperature and precipitated in methanol. The obtained white polymer beads were filtered, washed multiple times with deionized water, and vacuum-dried at 80 °C for 24 h, yielding 6FDA-durene polyimide (2.83 g, 88% yield). *δ*_H_ (400 MHz, CDCl_3_): 8.1–8.0 (2H, broad signal, 2 × ArH), 8.0–7.9 (4H, broad signal, 4 × ArH), and 2.1 (12H, singlet, 3 × CH_3_); (ATR-FTIR)/cm^−1^: 2930, 1788, 1713, 1345, 1251, 1179, 1114, and 977; GPC (CHCl_3_, RI)/Da M_n_ 61.00 kg/mol, and Mw 109.54 kg/mol and M_w_/M_n_ 1.80.

### 2.4. Preparation of PEG-MOF-Incorporated 6FDA-Durene-Based Mixed Matrix Membranes 

A matrix of 300 mg 6FDA-durene polymer was dissolved in 10 mL of chloroform and stirred for 1 h. For the creation of MMM-3, MMM-5, MMM-10, and MMM-15, PEG-MOF filler was dispersed in 3 mL of chloroform at concentrations of 3 wt%, 5 wt%, 10 wt%, and 15 wt% relative to the 6FDA matrix using ultrasonication and stirring. Subsequently, the dispersed filler solution was introduced to the 6FDA-durene solution and subjected to sonication to ensure uniform distribution of the MOF throughout the matrix. The resultant mixture was then poured into a Petri dish and allowed to air dry for 12 h at room temperature to form a membrane. To thoroughly eliminate the solvent, the prepared membrane was further dried at 80 °C in a vacuum oven. 

### 2.5. Characterization and Measurements

The membranes’ structures and physical properties were assessed using a ^1^H NMR Agilent 400-MR (400 MHz) instrument (Santa Clara, CA, USA), employing deuterated chloroform (CDCl_3_) and deuterium oxide (D_2_O) as solvents, with tetramethylsilane (TMS) as a reference for the internal deuterium lock. Fourier Transform Infrared (FTIR) spectroscopy was conducted using a PerkinElmer FT-IR Spectrum Two (Shelton, WA, USA). The membrane thickness was measured using a micrometer (Mitutoyo Model 547-201, Sakado, Japan). Thermal characteristics were determined through thermogravimetric analysis (TGA) using a Scinco TGA/N-1000 analyzer (Seoul, Republic of Korea) employing a nitrogen flow at a heating rate of 10 °C min^−1^. Morphological evaluation was carried out using wide-angle X-ray diffraction (WAXD) and scanning electron microscopy (SEM). WAXD spectra of the membranes were collected using a Rigaku HR-XRD Smart Lab diffractometer (Tokyo, Japan) at a scanning rate of 0.2° min^−1^ within a 2θ range spanning 5° to 50°, with a Cu-Kα X-ray source (λ = 1.54 Å). The BET surface area was determined through nitrogen adsorption experiments at 77 K using a Micromeritics ASAP 2020 HD88 instrument (Norcross, GA, USA). Gas separation measurements were conducted using the Time-lag instrument under constant volume–variable pressure conditions, as outlined in our prior study [36]. Detailed information regarding the instrumentation and characterization procedures is provided in the Supporting Information. 

## 3. Results and Discussion

### 3.1. Synthesis and Characterization of UiO-66-NH_2_ and UiO-66-NH_2_ (PEG-MOF) Containing PEG

**Figure 1:**
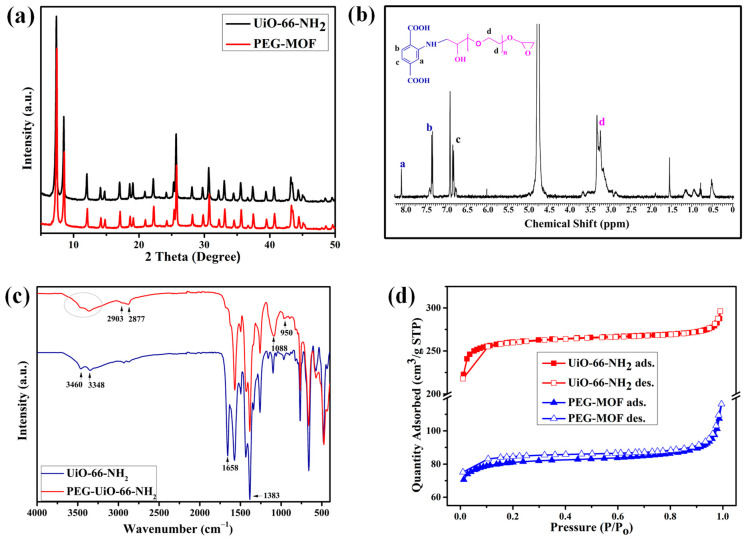
(**a**) PXRD graphs of UiO-66-NH_2_ and PEG-MOF, (**b**) ^1^H NMR spectra of the PEGDE-grafted MOF, (**c**) ATR-FTIR spectra of UiO-66-NH_2_ and PEG-MOF, and (**d**) N_2_ adsorption (solid line-filled symbol)/desorption (solid line-empty symbol) isotherms of UiO-66-NH_2_ and PEG-MOF.

Zirconium chloride was subjected to a reaction with 2-amino terephthalic acid, employing the test procedure outlined in existing literature [35]. The objective was to synthesize UiO-66-NH_2_, characterized as an amine-terminated metal–organic framework (MOF) as depicted in Scheme 1. The molecular structure of UiO-66-NH_2_ was confirmed using ^1^H NMR (Figure S1). The crystalline structure of the resultant UiO-66-NH_2_ was investigated using powder X-ray diffraction (PXRD). A thorough cross-reference was conducted by juxtaposing the PXRD findings with the corresponding X-ray diffraction analysis outcomes documented in the literature [23]. This comparative analysis unequivocally validated the congruence of the obtained patterns with those detailed in the literature (Figure 1a).

Following this, a reaction was carried out between UiO-66-NH_2_ and poly(ethylene glycol) diglycidyl ether (PEGDE) to incorporate ethylene oxide (EO) moieties into the UiO-66-NH_2_ structure to serve as CO_2_-solubilizing entities. This procedure yielded a PEG-grafted metal–organic framework (PEG-MOF) depicted in Scheme 1 [34]. 

The structural assessment of the synthesized PEG-MOF commenced by subjecting it to digestion using an aqueous solution of NaOD, yielding its ^1^H NMR spectra for detailed structural analysis (Figure 1b). The analysis revealed distinctive absorption peaks corresponding to Ar-H at the 6.5–7.5 ppm range, along with proton peaks attributable to the -CH- and -CH_2_ functionalities of PEG within the PEG-MOF, discernible at 1.3–3.75 ppm. This unequivocally confirmed the successful functionalization of the amine groups present in UiO-66-NH_2_ with PEGDE. 

Further validation of the structural integrity of both the UiO-66-NH_2_ MOF and the PEG-MOF was pursued through FTIR spectroscopy (Figure 1c). The distinctive peaks detected at 3460 cm^−1^ and 3348 cm^−1^ within the MOF spectrum were attributed to the asymmetric and symmetric stretching vibrations of the primary amine. Notably, the bands evident at 1658 cm^−1^ (asymmetric stretching) and 1383 cm^−1^ (symmetric stretching) were linked to the carboxyl groups originating from the 2-aminoterephthalic acid present in UiO-66-NH_2_. However, discernible distinctions emerged subsequent to the incorporation of PEGDE into the MOF. The broad absorption peaks spanning the 3200 cm^−1^ to 3600 cm^−1^ interval were attributed to the hydroxyl and secondary amine groups inherent to PEG-MOF. Furthermore, the methylene group derived from the PEGDE graft in UiO-66-NH_2_ exhibited dual peaks at 2914 cm^−1^ and 2875 cm^−1^, corresponding to the asymmetric and symmetric stretching vibrations. Of utmost significance, definitive proof of PEGDE grafting within the PEG-MOF was underscored by the emergence of distinctive peaks at 1088 cm^−1^ and 950 cm^−1^, resonating with the stretching vibration of the ether group (C-O-C) intrinsic to PEGDE [34,37]. 

Subsequently, an in-depth analysis of the crystalline structure of UiO-66-NH_2_ was executed through PXRD and the alterations in structure pre- and post-functionalization were scrutinized (Figure 1a). The diffraction peaks observed in UiO-66-NH_2_ exhibited alignment with those documented in the existing literature [38,39,40]. Notably, the PXRD spectra of PEG-MOF displayed a near-identical pattern to that of UiO-66-NH_2_. This substantiated that the crystallinity and fundamental framework of the MOF remained conserved, even subsequent to the grafting of PEGDE onto UiO-66-NH_2_. The PXRD spectra of PEG-MOF prominently showcased crystal peaks of high intensity, indicative of the MOF’s channel structure being extended over a significant distance, thus rendering it conducive for molecular transportation. 

Subsequently, the nitrogen adsorption and desorption characteristics of both UiO-66- NH_2_ and PEG-MOF were assessed at 77 K (Figure 1d). The determination encompassed the measurement of BET surface areas, revealing that PEG-MOF exhibited an area of 317.47 m^2^ g^−1^, which was notably lower in comparison to UiO-66-NH_2_ (1041.8 m^2^ g^−1^). This reduction in the surface area within PEG-MOF was ascribed to the partial occupation of the pores by neighboring PEG chains integrated into the MOF framework. In essence, the PEGDE units effectively utilized the vacant volume on the MOF’s surface. This outcome substantiated the successful grafting of PEGDE onto the UiO-66-NH_2_ MOF, thereby culminating in the synthesis of PEG-MOF. 

**Figure 2:**
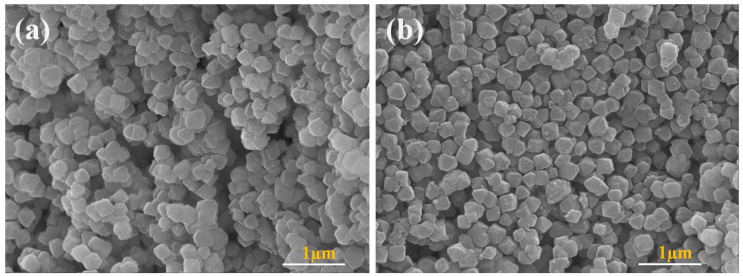
SEM images of (**a**) UiO-66-NH_2_ and (**b**) PEG-MOF.

The morphologies and particle dimensions of UiO-66-NH_2_ and PEG-MOF were additionally subjected to scrutiny through SEM, as depicted in Figure 2. The findings unequivocally revealed that the incorporation of PEGDE did not induce substantial alterations in the morphological characteristics. 

### 3.2. Fabrication and Characterization of MMMs

#### 3.2.1. Fabrication of MMMs

**Figure 3:**
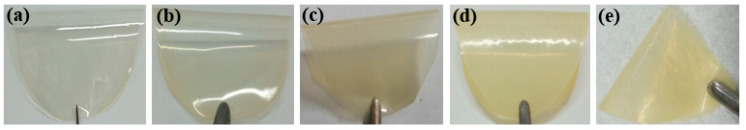
Images of PEG-MOF-containing MMMs with different concentrations: (**a**) 0 wt%, (**b**) 3 wt%, (**c**) 5 wt%, (**d**) 10 wt%, and (**e**) 15 wt%.

Solution casting was executed employing CHCl_3_ as the solvent to incorporate PEG-MOF particles into the 6FDA-durene-based PI support at diverse concentrations spanning 3 wt%, 5 wt%, 10 wt%, and 15 wt%. This strategy was employed to fabricate mixed matrix membranes (MMMs). The resulting MMMs uniformly exhibited flexibility and were individually designated as MMM-3, MMM-5, MMM-10, and MMM-15 (Figure 3). 

#### 3.2.2. Morphological Analysis Using WAXD and SEM 

**Figure 4:**
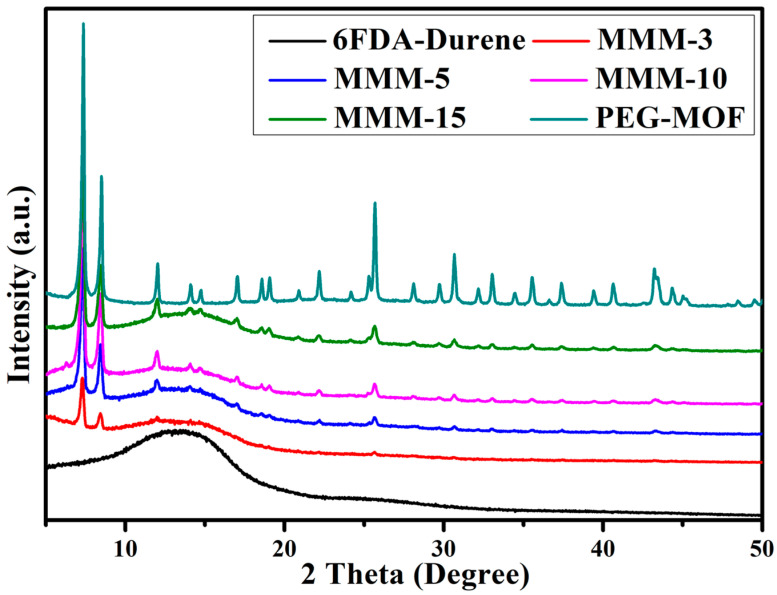
XRD patterns of the pristine 6FDA-durene membrane, PEG-MOF, and the MMMs with varying PEG-MOF contents.

Alterations in the morphological attributes of the MMMs subsequent to the integration of PEG-MOF, a PEGDE-grafted MOF, were scrutinized via wide-angle X-ray diffractometry (WAXD) (Figure 4). In the pristine state of the 6FDA-durene-PI (prior to PEG-MOF incorporation), a broad peak materialized at 13.6°, aligning with the distinctive amorphous signature of 6FDA-durene.

Across all MMMs incorporating PEG-MOF, the aforementioned broad diffraction peak consistently manifested at an identical position; however, its intensity notably diminished (Figure 4). This attenuation in peak intensity was rationalized by the incorporation of the MOF particles into the polymer support [22]. In marked contrast, distinct peaks characteristic of the rigid crystalline configuration of the introduced MOF exhibited an augmented intensity, aligning with an increase in MOF content. This unequivocally underscored the preservation of the innate crystalline structure of the incorporated filler particles within the MMMs. Such a trend finds commonality within the diffraction spectra of various other MMM variants [41,42,43]. 

**Figure 5:**
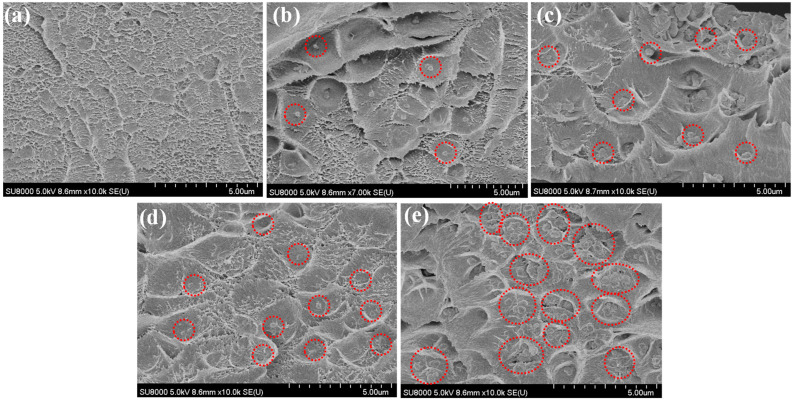
SEM cross-sectional images of the MMM with different PEG-MOF contents (**a**) 6FDA-durene, (**b**) MMM-3, (**c**) MMM-5, (**d**) MMM-10, and (**e**) MMM-15 {PEG-MOF filler particles in the polymer matrix indicated by red dotted circles}.

The microstructural attributes of the acquired MMMs were subjected to further analysis in relation to the content of PEG-MOF, utilizing their SEM cross-sectional imagery as a basis. These findings were then juxtaposed with the microstructural characteristics evident within the unmodified 6FDA-durene-based membrane (Figure 5). When examining the cross-sectional views of both 6FDA-durene and all MMMs, no instances of defects were discernible (Figure 5a). In contrast, the dispersion of MOF particles within the 6FDA-durene polymer matrix exhibited variance contingent on the MOF content (Figure 5b–e). A homogeneous distribution of PEG-MOF in MMMs was observed in the SEM-EDX mapping images (Figure S2). Specifically, the PEG-MOF particles exhibited uniform dispersion within MMM-3 (Figure 5b), MMM-5 (Figure 5c), and MMM-10 (Figure 5d). However, in the case of MMM-15 (Figure 5e), some aggregations of PEG-MOF particles were observable. 

Broadly speaking, the even dispersion of MOF particles within a polymer matrix facilitates the rapid diffusion of gas molecules through the MOF structure’s pores. This, in turn, engenders heightened efficacy in molecular transport and, consequently, improved gas permeability [19,20,24,26]. Conversely, in cases where the membrane structure consists of particle aggregates, the pores encircling the MOF-polymer interface might experience blockage, or the polymer could undergo densification. These effects could potentially culminate in a decrease in gas separation efficiency [11]. 

Consequently, an assessment of the morphological attributes of the MMMs and the impact incurred by the introduction of PEG-MOF was conducted, relying on the insights drawn from the XRD and SEM analyses. Intriguingly, across all MMMs, no instances of defects were identified, irrespective of the PEG-MOF content. Nonetheless, this exploration enabled the identification of an optimal MOF content, one that fosters a more homogeneous distribution of MOF particles, thereby translating into enhanced gas separation efficacy. 

### 3.3. Thermal Properties of the MMMs

Thermogravimetric analysis (TGA) was conducted to investigate the impact of PEG-MOF on the thermal characteristics of the resulting MMMs (Figure S3). In the TGA graph of UiO-66-NH_2_, a noticeable weight loss transpired due to moisture evaporation from the surface at temperatures below 100 °C. This was followed by an additional weight loss of around 134 °C attributed to the evaporation of absorbed moisture or the solvent (DMF). Beyond 300 °C, a third weight loss, attributed to MOF decomposition, was observed (Figure S3a) [22,23]. Comparatively, the initial decomposition temperature of PEG-MOF exceeded that of UiO-66-NH_2_ due to the grafting of PEG (Figure S3a).

Subsequently, the thermal characteristics of the PEG-MOF-incorporated MMMs were scrutinized and compared against those acquired from the pristine 6FDA-durene-based PI (Figure S3b). The initial weight loss observed in the MMMs proved to be smaller than that exhibited by the pristine 6FDA-durene membrane. Notably, this tendency showed an increase with rising PEG-MOF content. This phenomenon was ascribed to the fact that PEG-MOF decomposition transpired at temperatures lower than those required for the degradation of 6FDA-durene. Across the various MMMs containing distinct proportions of PEG-MOF, the onset of MOF decomposition manifested at 300 °C, a value notably lower than that of 6FDA-durene. In contrast, the weight reduction linked to the primary polymer’s decomposition was consistent across all MMMs, manifesting at nearly the same temperature. 

### 3.4. Gas Separation Performance of MMMs

The gas separation efficiency of the MMMs incorporating the PEG-MOF—a UiO-66-NH2 variant with PEG grafting—was evaluated using constant-volume and variable-pressure techniques at a temperature of 30 °C and a pressure of 1 atm (Table 1 and Table 2). Subsequent to the assessment, the acquired findings were juxtaposed with the data derived from the 6FDA-durene-PI membrane.

The permeability of the MMMs containing PEG-MOF displayed an ascending trend corresponding to the augmented PEG-MOF content (Table 1). This trend suggested that gas molecules traversed the MOF pores and polymer matrix in a sequential manner. Particularly noteworthy was the significantly elevated permeability of CO_2_ observed across all membranes in comparison to other gases. This outcome was attributed to the diminutive kinetic diameter of CO_2_, coupled with its potent interaction with PEG. As a consequence, an enhanced solubility–selectivity emerged (Table 2). The gas separation performance of an MMM comprising 10 wt% of unaltered (without PEG) MOF was further compared. In particular, the permeability of all the gases increased for the molecules with smaller kinetic diameters compared to their behavior in the pristine 6FDA-durene PI (Table 1). This increase is attributed to the increased diffusivity of gas molecules through the porous channels in the membrane due to the MOFs (Table 2). Although the permeabilities of the MMMs with PEG-modified MOFs were lower than in UiO-66-NH_2_-MMM, significant improvements in CO_2_/N_2_ and CO_2_/CH_4_ selectivities compared to their behavior in UiO-66-NH_2_-MMM were observed due to the increased solubility–selectivity obtained from the CO_2_-philic PEG groups after modification of the MOF.

Generally, a gas’s permeability in membranes is influenced by its kinetic diameter. The kinetic diameters of the gases used in the measurements are as follows: CH_4_ (3.8 Å) > N_2_ (3.64 Å) > CO_2_ (3.3 Å). It was discerned that as the kinetic diameter of a gas diminished, its permeability within a membrane augmented [44]. In simpler terms, CO_2_, characterized by a smaller kinetic diameter, displayed heightened diffusivity in comparison to the other gases (Table 2). 

The selectivity of each membrane was determined by calculating the ratio of permeabilities for two distinct gases within them. Notably, the CO_2_ selectivity of the MMMs in relation to N_2_ and CH_4_, both nonpolar gases, exhibited a superior value in comparison to the 6FDA polymer. This enhancement stemmed from the substantial solubility of the PEG functional groups integrated within the MOF, particularly within the CO_2_ environment. 

**Figure 6:**
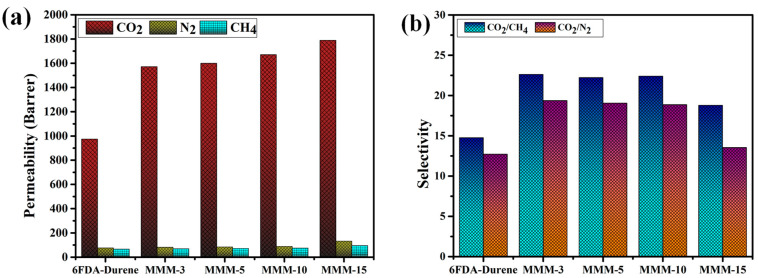
(**a**) Permeability and (**b**) selectivity of the MMMs with different MOF contents.

Out of all the membranes, the MMM featuring a MOF content of 10 wt% demonstrated the most favorable gas separation performance, excelling in both permeability and selectivity (Figure 6a,b). With a higher MOF content (15 wt%), the permeability exhibited an increase, yet both CO_2_/N_2_ and CO_2_/CH_4_ selectivities experienced a decline. This phenomenon could be attributed to the emergence of non-selective pores brought about by MOF aggregation within this particular MMM, a conclusion substantiated by earlier morphological analysis findings [11,45,46]. 

It is important to highlight that the augmentation in permeability observed in the MOF-incorporated MMMs primarily stems from the enhancement of their diffusion coefficients.

Nevertheless, in the context of this study, the solubility–selectivity traits of the PEG constituents took precedence, shaping the overarching diffusion behavior that consequently bolstered the permeability of the MMMs. This interpretation finds further reinforcement in the preceding BET analysis outcomes. Within the PEG-MOF structure, the pores were partially occupied by CO_2_-philic PEG components, contributing to a decrease in the BET surface area and the overall pore volume. 

As per the existing literature, PEG components exhibit remarkable flexibility, enabling them to efficiently interface with the MOF surface and even permeate the MOF pores [47,48,49,50]. This phenomenon is particularly accentuated within UiO-66-based MOFs and PEG systems, where the presence of robust hydrogen bonds between the MOF and the oligomers is notable [51,52,53].

Furthermore, the microstructural examination conducted through SEM corroborated the absence of any defects within these MMMs. This observation underscores the nearly flawless interfacial compatibility prevailing between the PEG-MOF and the filler matrix within these membranes. 

### 3.5. Permeability Versus Selectivity of the MMMs 

**Figure 7:**
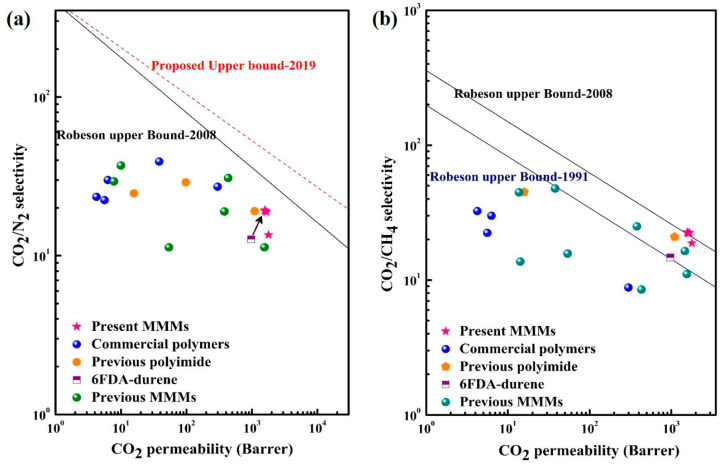
Comparison of separation performances between the PEG-MOF-based MMMs and other polyimides, commercial polymers, and other MMMs concerning the 2008 Robeson upper bounds. (**a**) CO_2_/N_2_ and (**b**) CO_2_/CH_4_. Table S2 in the Supplementary Material contains information on each referenced membrane.

The permeability and selectivity data of the PEG-MOF-containing mixed matrix membranes (MMMs) developed in the present study are presented in Figure 7. These results have been juxtaposed with the 2008 Robeson upper bounds [5,6,46] to assess their separation performance in terms of CO_2_/N_2_ and CO_2_/CH_4_. Inclusion for comparative analysis comprises data from previously documented polyimide-based membranes [54,55,56], mixed matrix membranes incorporating well-established MOFs (UiO-66, ZIF-8, UiO-66-NH_2_) [44,57,58,59,60], as well as commercial polymers (Pebax, polyurethane, cellulose acetate, polysulfone, and polycarbonate) [59,61,62].

The MMM developed in this study demonstrated superior performance compared to the 6FDA-durene membrane and other polyimide membranes such as 6FDA-DAM [56], PI-BAFL-6FDA [55], and 6FDA-MDA [54], in addition to the aforementioned commercial membranes. Furthermore, the separation efficiency of these MMMs surpassed that of previously reported 6FDA-durene-based MMMs containing UiO-66-NH_2_ [58] and ZIF-8 [44]. 

According to Figure 7a, the performance of the pristine 6FDA-durene membrane falls considerably below the 2008 upper bound established for CO_2_-N_2_ separation. Conversely, all recently developed mixed matrix membranes (MMMs) exhibited substantial enhancements, progressively nearing the 2008 Robeson upper bound. Notably, MMM-3, MMM-5, and MMM-10 demonstrated a remarkable superiority not only over the unmodified 6FDA-durene membrane but also over the aforementioned commercial polymers, previously reported polyimides [54,55,56,59,61,62], and existing MMMs [44,57,58,59,60] in terms of CO_2_-CH_4_ separation. This achievement is vividly illustrated by their capacity to transcend the 2008 Robeson upper bound, as illustrated in Figure 7b.

Among the array of prepared MMMs, MMM-15 stands out, revealing a CO_2_ permeability of 1789.50 Barrer and a CO_2_-CH_4_ selectivity of 18.78. While it falls below the 2008 upper bound, it unquestionably showcased superior performance compared to the reported MMMs. This observation underscores the overall excellence in permeability and selectivity exhibited by all the prepared MMMs, rendering them compelling prospects for industrial membrane applications. 

## 4. Conclusions

In this study, we started by synthesizing UiO-66-NH_2_ nanoparticles, then covalently modified them through the grafting of a CO_2_-philic PEGDE unit. These tailored MOFs, referred to as PEG-MOFs, were subsequently integrated into a 6FDA-durene matrix at varying weight percentages (3 wt%, 5 wt%, 10 wt%, and 15 wt%) using a solution-casting technique to craft mixed matrix membranes (MMMs). Extensive characterization encompassing XRD, SEM, and TGA analyses was conducted on the fabricated membranes. In contrast to the unmodified MOF, the PEG-MOF exhibited an increased presence of organic segments, promoting superior interfacial compatibility between the polymer matrix and MOFs. Notably, up to a loading of 10 wt% PEG-MOF, the polymer matrix and filler demonstrated a synergistic affinity, yielding a defect-free structure for the MMM, a fact confirmed by SEM outcomes.

The remarkable compatibility between the PEG-grafted MOF and the polymer matrix, combined with the presence of CO_2_-philic PEG units, imparted a noteworthy enhancement in the gas separation performance across all the MMMs developed in this study. With an augmented PEG-MOF content, CO_2_ permeability and CO_2_/N_2_ as well as CO_2_/CH_4_ selectivity exhibited a progressive increase. Particularly notable are the MMMs—specifically, MMM-3, MMM-5, and MMM-10—incorporating 3 wt%, 5 wt%, and 10 wt% PEG-MOF loading, which surpassed the 2008 Robeson upper bound for CO_2_/CH_4_ separation. Importantly, when juxtaposed with the unmodified 6FDA-durene polymer membrane (P(CO_2_) = 973.90 Barrer, CO_2_/N_2_ = 12.71, and CO_2_/CH_4_ = 14.76), the MMM incorporating 10 wt% PEG-MOF (MMM-10) boasted exceptional CO_2_ permeability (P(CO_2_) = 1671.00 Barrer) along with superior CO_2_ selectivity over N_2_ and CH_4_ (CO_2_/N_2_ = 18.86 and CO_2_/CH_4_ = 22.40). Emerging as the top-performing membrane, it not only surpassed the 2008 Robeson upper bound for CO_2_/CH_4_ separation but also approached the upper bound for CO_2_/N_2_ separation, positioning it as a highly promising candidate for efficient CO_2_ extraction from flue gases.

Furthermore, the TGA analysis validated the robust thermal stability of these membranes—a vital prerequisite for the prospective commercialization of gas separation membranes. Based on the findings of this investigation, we envision that through a well-devised interface modification strategy for MOF fillers within MMMs, it is conceivable to obtain commercial-grade membranes exhibiting robust gas separation performance.

## Figures and Tables

**Table 1:** Gas permeability and selectivity of the fabricated membranes.

Membrane	^a^ Permeability (Barrer)			Selectivity, α	
	CO_2_	N_2_	CH_4_	CO_2_/N_2_	CO_2_/CH_4_
6FDA-durene	973.90	76.60	66.00	12.71	14.76
UiO-66-NH_2_ (10 wt%)-MMM	2558.92	156.38	153.89	16.36	16.63
MMM-3	1572.13	81.20	69.50	19.36	22.62
MMM-5	1600.00	84.00	72.00	19.05	22.22
MMM-10	1671.00	88.60	74.60	18.86	22.40
MMM-15	1789.50	132.30	95.30	13.53	18.78

^a^ Permeability (Barrer), where 1 Barrer = 10^−10^ [cm^3^(STP)·cm]/(cm^2^.s.cmHg).

**Table 2:** Gas diffusion coefficient, solubility coefficient, diffusivity selectivity, and solubility selectivity of each MMM.

Membrane	Diffusivity, D ^a^			Diffusivity Selectivity		Solubility, S ^b^			Solubility Selectivity	
	CO_2_	N_2_	CH_4_	CO_2_/N_2_	CO_2_/CH_4_	CO_2_	N_2_	CH_4_	CO_2_/N_2_	CO_2_/CH_4_
6FDA-durene	18.55	15.40	3.00	1.20	6.18	0.53	0.05	0.22	10.60	2.41
UiO-66-NH_2_ (10 wt%)-MMM	43.27	30.16	8.55	1.43	5.06	0.59	0.05	0.18	11.80	3.28
MMM-3	18.60	16.10	3.54	1.15	5.25	0.85	0.05	0.19	17.00	4.47
MMM-5	14.90	13.60	8.20	1.09	1.82	1.07	0.06	0.09	17.83	11.89
MMM-10	12.70	12.50	7.75	1.02	1.64	1.31	0.07	0.10	18.71	13.10
MMM-15	25.20	12.05	11.30	2.09	2.23	0.71	0.10	0.08	7.10	8.88

^a^ D (10^−8^ cm^2^ s^−1^); ^b^ S in [cm^3^ (STP)]/(cm^3^ cmHg).

## Data Availability

Data are contained within the article.

## Supplementary Materials

The following supporting information can be downloaded at: https://www.mdpi.com/article/10.3390/polym15224442/s1, Figure S1: ^1^H NMR spectra of (a) UiO-66-NH_2_, (b) PEGDE and (c) 6FDA-durene; Figure S2: EDX elemental mapping images of (a) MMM-3, (b) MMM-5, (c) MMM-10, and (d) MMM-15; Figure S3: Thermal characteristics with respect to the PEG-MOF content (a) UiO-66-NH_2_ and PEG-MOF and (b) MMMs; Table S1: BET surface area and pore volume of MOF and modified MOF; Table S2: Gas separation properties of various gas separation membranes.
