# Supplementary material for: Developing Mixed Matrix Membranes with Good CO2 Separation Performance Based on PEG-Modified UiO-66 MOF and 6FDA-Durene Polyimide
Source: Polymers (Basel). 2023 Nov 17;15(22):4442. doi: 10.3390/polym15224442 (PMC10674161; doi:10.3390/polym15224442)
Supplement: Supplementary file 1 [file polymers-15-04442-s001.zip › polymers-2659349-supplementary.pdf]

# *Supporting Information for*

# Developing mixed matrix membranes with good $CO_2$ separation performance based on PEG-modified UiO-66 MOF and 6FDA-durene polyimide

**Kavya Adot Veetil [1,2,†], Asmaul Husna [1,2,†], Md. Homayun Kabir [1,2], Insu Jeong [1,2], Ook Choi [1,2], Iqubal Hossain [1,2], Eun kyu Sun [1,2] and Tae-Hyun Kim [1,2,*]**

1 Organic Material Synthesis Laboratory, Department of Chemistry, Incheon National University, Incheon 406-772, Republic of Korea
2 Research Institute of Basic Sciences, Core Research Institute, Incheon National University, 119 Academy-ro, Yeonsu-gu, Incheon 22012, Republic of Korea
* Correspondence: tkim@inu.ac.kr
† These authors contributed equally to this work.

**This file includes:**

Experimentals

Figure S1 & S2 & S3

Table S1 & S2

**Experimentals**

S1. Gas Permeation Measurement: Constant-Volume/Variable-Pressure Measurements

S 1.1. Single gas separation analysis

Gas permeability measurements of pure gases were evaluated using a vacuum-applied time-lag instrument based on a constant-volume/variable-pressure method. All of the experiments were carried out at a feed pressure of 1 bar and a temperature of 30 °C. To remove all residual gases, before the measurement, both upstream and downstream were evacuated thoroughly to below $10^{-5}$ Torr ($1.33\times10^{-8}$ bar) until the readout appeared to be zero. The downstream volume was found to be 57 $cm^3$ by calibration using a Kapton membrane. A Baraton transducer (MKS; Model No. 626B02TBE) with full scales of 10,000 and 2 Torr was used to measure the upstream and downstream pressures respectively. The permeate side pressure was recorded as a function of time using a transducer. The permeability coefficient was obtained from the linear slope of the downstream pressure versus a time plot (dp/dt) according to the following equation:

$$P = \frac{273\ \mathrm{K}}{76\ \mathrm{cmHg}} \times \frac{Vl}{ATp_0} \times \frac{dp}{dt} \qquad (1)$$

Where P is the permeability expressed in barrer (1 Barrer = 10−10 [cm3 (STP) cm cm−2 s −1 cm Hg−1 ], V (cm3 ) is the downstream volume, l (cm) is the membrane thickness, A (cm2 ) is the effective area of the membrane, T (K) is

the measurement temperature, $p_o$ (Torr) is the pressure of the feed gas in the upstream chamber, and dp/dt is the rate of the pressure change under a steady state. The permeation tests were repeated at least three times for each gas and the standard deviation from the mean values of the permeabilities was within ca. 3%. The sample-to-sample reproducibility was high and within 3%. The effective membrane areas were 1.13 $cm^2$. The ideal perm-selectivity, $\alpha$A/B, of the membrane for a pair of gases (A and B) is defined as the ratio of the individual gas permeability coefficients:

$$\alpha_{\frac{A}{B}} = \frac{PA}{PB} \qquad (2)$$

The diffusivity and solubility were obtained from the time-lag (θ) value according to the following equations:

$$D = \frac{l^2}{6\theta} \qquad (3)$$

$$S = \frac{P}{D} \qquad (4)$$

Where, D (cm2 s -1) is the diffusivity coefficient, l is the membrane thickness (cm), and θ is the time lag (s), as obtained from the intercept of the linear steady-state part of the downstream pressure versus a time plot. The solubility, S, was calculated from Equation (4) with the permeability and diffusivity obtained from Equation (3) and (4).

(a)

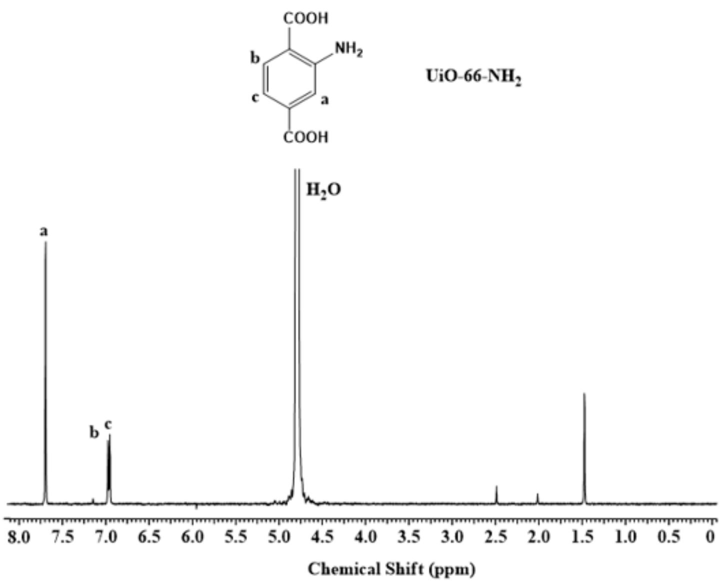
COOH
NH2
b
c
a
COOH
UiO-66-NH2
H2O
a
b
c
8.0 7.5 7.0 6.5 6.0 5.5 5.0 4.5 4.0 3.5 3.0 2.5 2.0 1.5 1.0 0.5 0
Chemical Shift (ppm)

(b)

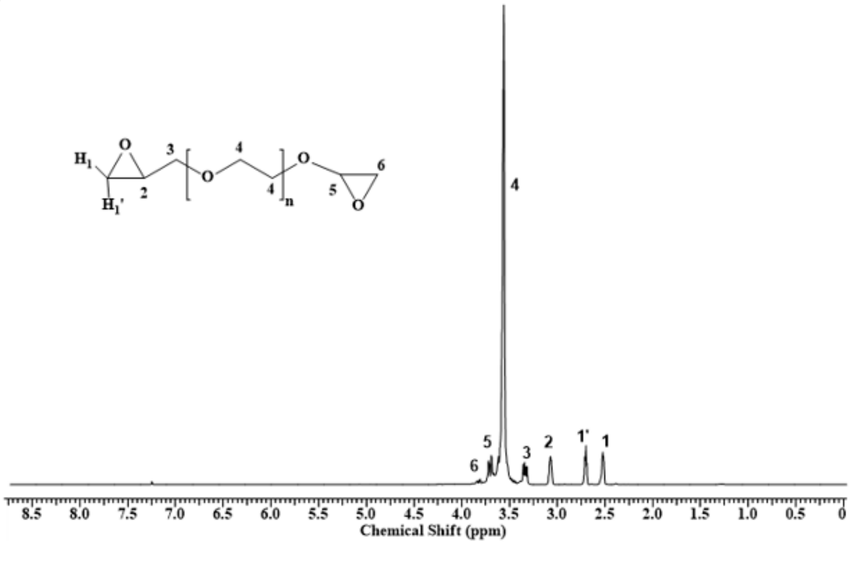
H1
H1'
O
2
3
4
4
n
O
O
5
6
O
4
6
5
3
2
1'
1
8.5 8.0 7.5 7.0 6.5 6.0 5.5 5.0 4.5 4.0 3.5 3.0 2.5 2.0 1.5 1.0 0.5 0
Chemical Shift (ppm)

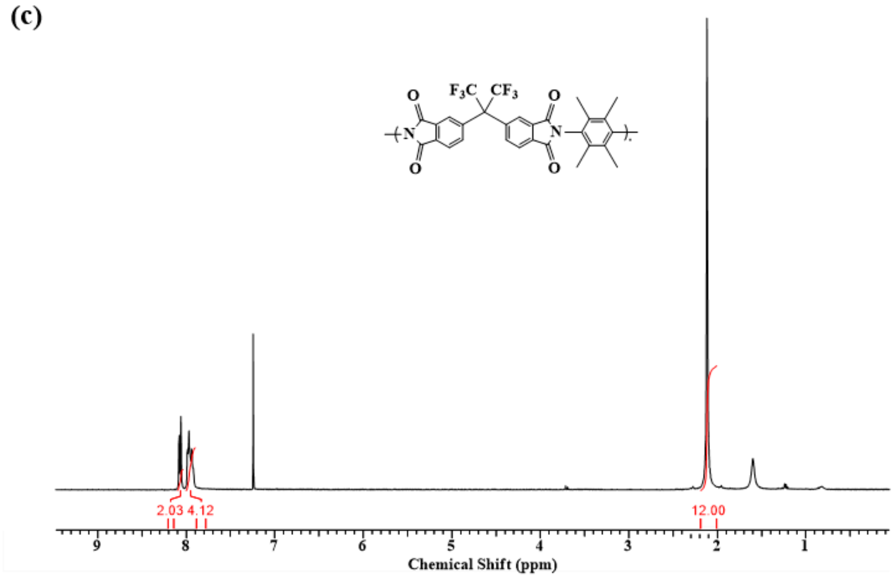


**Figure S1.** $^{1}H$ NMR spectra of (a) UiO-66-$NH_2$, (b) PEGDE and (c) 6FDA-durene

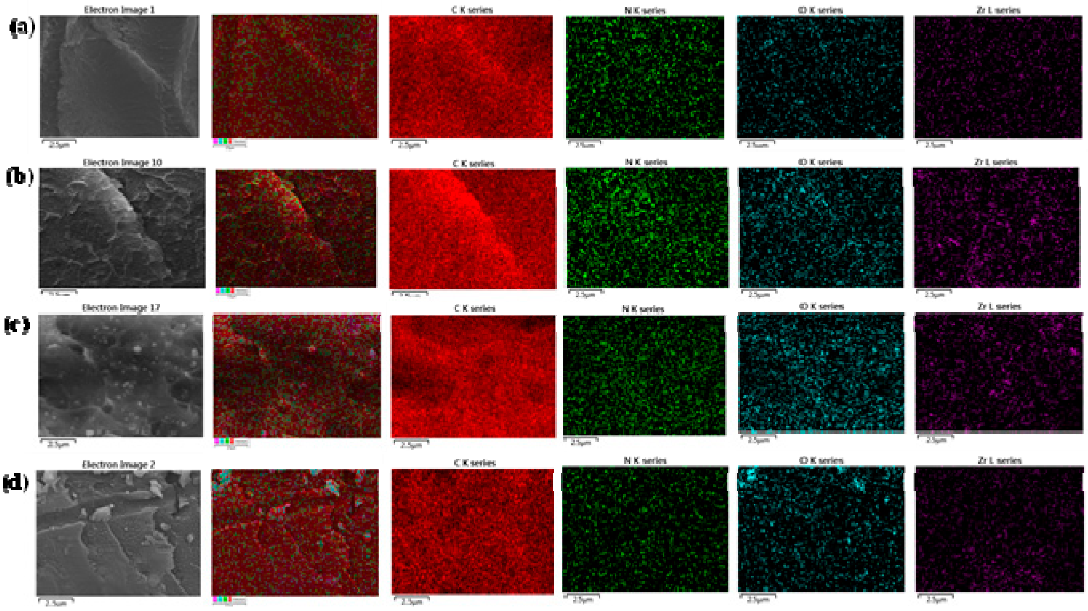


**Figure S2.** EDX elemental mapping images of (a) MMM-3, (b) MMM-5, (c) MMM-10, and (d) MMM-15

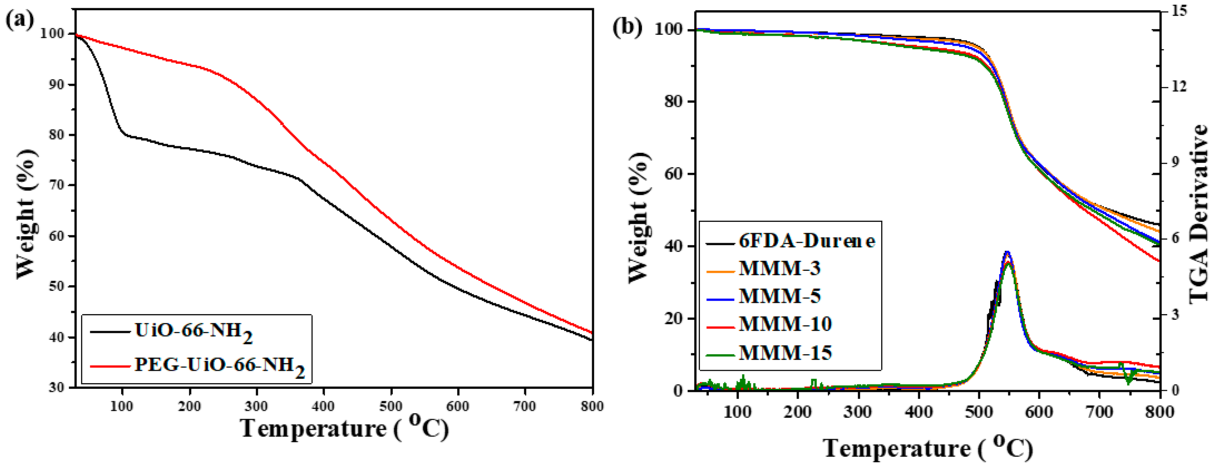


**Figure S3.** Thermal characteristics with respect to the PEG-MOF content (a) UiO-66-$NH_2$ and PEG-MOF and (b) MMMs

**Table S1.** BET surface area and pore volume of MOF and modified MOF

| Name | Surface area m²/g | Pore volume |
|---|---|---|
| UiO-66-$NH_2$ | 1041.80 | 0.458 |
| PEG-MOF | 317.47 | 0.179 |

**Table S2.** Gas separation properties of various gas separation membranes

| Filler | Polymer | Loading (wt %) | Measurement conditions | $P_{CO2}$ (barrer) | $CO_2/CH_4$ selectivity | $CO_2/N_2$ selectivity | Ref. |
|---|---|---|---|---|---|---|---|
| | | | PI membranes | | | | |
| --- | 6FDA-MDA | --- | | 15.8 | 44.9 | 24.7 | [1] |
| --- | PI-BAFL-6FDA | --- | 25 °C at 76 cmHg. | 98 | ----- | 29 | [2] |
| --- | 6FDA-DAM-PI | --- | 15 psi and 35 °C | 1100 | 20.9 | 19.1 | [3] |
| | | | Commercial Polymers | | | | |
| --- | polyurethane | --- | 4 bar | 38.4 | -- | 39.2 | [4] |
| --- | Pebax | --- | 6 bar; 298 K | 299 | 8.8 | 27.2 | [5] |
| --- | Cellulose acetate | --- | | 6.3 | 30 | 30 | [6] |
| --- | Polysulfone | --- | | 5.6 | 22.4 | 22.4 | |
| --- | Polycarbonate | --- | | 4.23 | 32.5 | 23.5 | |
| | | | MMMs | | | | |
| ZIF-8 | DMPBI-BuI | 30 | 35 oC, 20 bar | 53.9 | 15.7 | 11.3 | [7] |
| UiO-66-NH2 | 6FDA-ODA | 25 | 10 bar, 35 °C | 13.7 | 44.7 | --- | [8] |
| UiO-66-NH2 | 6FDA–Durene | 20 | 1 bar, 35 °C | 1470 | 16.4 | --- | [8] |
| UiO 66-NH2 | Matrimid 9725 | 30 | 9 bar; 308 K; equimolar CO2 and CH4 | 37.9 | 47.7 | --- | [5] |
| ZIF-8 | PU | 30 | 4 bar | 14.2 | 13.7 | --- | [5] |
| ZIF-8 | Polydopamine-polyimide | 7 | 1 bar; 308 K | 380 | 25 | 19 | [5] |
| ZIF-8 | Pebax | 10 | 6 bar; 298 K | 433 | 8.5 | 30.9 | [5] |
| ZIF-8 | 6FDA-durene | 33.3 wt % | 35 °C and 3.5 atm | 1552.9 | 11.07 | 11.3 | [9] |
| UiO-66 | Matrimid | 10 | 4 bar, 37 °C | 7.8 | --- | 29.4 | [10] |
| Azo-UiO-66 | Matrimid | 10 | 4 bar, 37 °C | 10 | --- | 37 | |
| **PEG-UiO-66-$NH_2$** | 6FDA–Durene | 0 | 1 bar, 30 °C | 973.9 | 14.7 | 12.7 | This work |
| | | 3 | | 1572.13 | 22.6 | 19.4 | |
| | | 5 | | 1600 | 22.2 | 19.1 | |
| | | 10 | | 1671 | 23.4 | 19.0 | |
| | | 15 | | 1789.5 | 18.1 | 14.0 | |

**References:**

[1] Zhang, C.; Cao, B.; Coleman, M. R.; Li, P. Gas Transport Properties in (6FDA-RTIL)-(6FDA-MDA) Block Copolyimides. *J. Appl. Polym. Sci.* **2015**, *133*.

[2] Kazama, S.; Teramoto, T.; Haraya, K. Carbon Dioxide and Nitrogen Transport Properties of Bis(Phenyl)Fluorene-Based Cardo Polymer Membranes. *J. Membr. Sci.* **2002**, *207*, 91–104.

[3] Kim, K. J.; Chae, Y.; An, S. J.; Jo, J. H.; Park, S.; Chi, W. S. Microphase-Separated Morphology Controlled Polyimide Graft Copolymer Membranes for CO2 Separation. *Sep. Purif. Technol.* **2023**, *304*, 122315.

[4] Rodrigues, M. A.; Ribeiro, J. de; Costa, E. de; Miranda, J. L.; Ferraz, H. C. Nanostructured Membranes Containing UiO-66 (Zr) and MIL-101 (Cr) for O2/N2 and CO2/N2 Separation. *Sep. Purif. Technol.* **2018**, *192*, 491–500.

[5] Prasetya, N.; Himma, N. F.; Sutrisna, P. D.; Wenten, I. G.; Ladewig, B. P. A Review on Emerging Organic-Containing Microporous Material Membranes for Carbon Capture and Separation. *J. Chem. Eng.* **2020**, *391*, 123575.

[6] Wu, D.; Hou, R.; Yi, C.; Smith, S. J. D.; Fu, J.; Ng, D.; Doherty, C. M.; Mulder, R. J.; Xie, Z.; Hill, M. R. Enhancing Polyimide-Based Mixed Matrix Membranes Performance for CO2 Separation Containing PAF-1 and P-DCX. *Sep. Purif. Technol.* **2021**, *268*, 118677.

[7] Ma, C.; Urban, J. J. Hydrogen-bonded Polyimide/Metal-organic Framework Hybrid Membranes for Ultrafast Separations of Multiple Gas Pairs. *Adv. Funct. Mater.* **2019**, *29*.

[8] Liu, B.; Li, D.; Yao, J.; Sun, H. Improved CO2 Separation Performance and Interfacial Affinity of Mixed Matrix Membrane by Incorporating UiO-66-PEI@[bmim][Tf2N] Particles. *Sep. Purif. Technol.* **2020**, *239*, 116519.

[9] Wijenayake, S. N.; Panapitiya, N. P.; Versteeg, S. H.; Nguyen, C. N.; Goel, S.; Balkus, K. J.; Musselman, I. H.; Ferraris, J. P. Surface Cross-Linking of ZIF-8/Polyimide Mixed Matrix Membranes (MMMs) for Gas Separation. *Ind. Eng. Chem. Res.* **2013**, *52*, 6991–7001.

[10] Prasetya, N.; Donose, B. C.; Ladewig, B. P. A New and Highly Robust Light-Responsive Azo-UiO-66 for Highly Selective and Low Energy Post-Combustion CO2 Capture and Its Application in a Mixed Matrix Membrane for CO2/N2 Separation. *J. Mater. Chem. A* **2018**, *6*, 16390–16402.
